# Dollars and Ideas

**Published:** 1881-06

**Authors:** 


### DOLLARS AND IDEAS.

A DOLLAR buys the poor man a dinner, and he passes it off. Tomorrow the same dollar does the same thing for some other man, and so it goes on, passing from hand to hand, feeding multitudes; and, so far from growing less in value, it is brightened by the handling, and, at the end of months and years of service, is worth as much as ever, while its very glistening makes it more tempting than one blackened by age and disuse.

"Every new idea is worth a silver dollar, young gentlemen," was a favorite stimulus to study, with our loved and venerated teacher, Ebenezer Sharp, long since in his Christian grave, and whose memory we bless.

Later on, we elsewhere learned, that there was a striking and practical resemblance between good ideas and good dollars; both grew more shining by use, for rust corrupted not, and disuse did not canker them away. Genuine dollars, like genuine ideas, increase the sum total, by circulation, by being passed around. Let us drop the dollar, and take up the idea. Truth is used by conversation and writing, and the oftener it is used thus, the brighter does it become, the more readily can it be applied, and the wider will be the range of uses which can be made of it. That which makes all the difference between the empty head and the crowded one, between a full man and a fool, is the facility with which things known can be made use of. It is not the mere fact of having money on hand, that makes a successful operator in Wall-street; it is the having it at command at an hour's notice, with the frequent judicious use of it: and that very use gives more power.

We take up a dollar, coined in the infancy of our Republic ; the man who moulded it has been dead for half a century. And yet, how many throbs of gratification has it excited, as it has been laid in the hand ; and how many a hungry mouth has it fed ; and yet it is as good as ever, and may continue to gladden the joyless, and feed the hungry, for a hundred years to come. But there is a remarkable difference between a good idea and a good dollar. The dollar is always received with pleasure ; but it is parted with, with a pang. But a good idea, well coined in words, gratifies him who gives, as well as him who receives.

One sterling truth, clothed in words of vigor, keenness, or sweetness, spoken or printed, passes from mouth to mouth, from paper to paper, from page to page, and will happify a million hearts. The dollar can be used by only one at a time, and but at one place in the same instant ; but the mental coin, by the art of all arts, can be used by multitudes, in all lands, at the same instant, distilling its sweetness on both giver and receiver everywhere, to be perpetuated for generations yet unborn.

The responsibilities of the press, of a writer, how amazing, how imperative, in the great contest of ages, between ignorance and error ! And the privilege of an ability to clothe our ideas in words that burn or bless ; in resistless power, or in honied sweetness, to wake up the sleepy and the lazy, or help up the weary, the lonely, or desponding, how high, how dear!

---

### FINGER NAILS

Grow out about three times a year ; they should be trimmed with scissors once a week, not so close as to leave no room for the dirt to gather, for then they do not protect the ends of the fingers, as was designed by nature ; besides, if trimmed too close at the corners, there is danger of their growing into the flesh, causing inconvenience, and sometimes great pain.

The collections under the ends of the nails should not be removed by anything harder than a brush or a soft piece of wood : nor should the nails be scraped with a penknife or other metallic substance, as it destroys the delicacy of their structure, and will at length give them an unnatural thickness. We are not favorably impressed as to the cleanliness of a person who keeps his nails trimmed to the quick, as it is often done to prevent dirt gathering there; whereas, if a margin were allowed, it would be an index to the cleanliness of the hands, from which the collections under the finger nails are made. Leave a margin, then, and the moment you observe that these collections need removal, you may know that the hands need washing, when they and the nails are both cleaned together.

Most persons are familiar with those troublesome bits of skin which loosen at the root of the finger nails; it is caused by the skin adhering to the nail, which, growing outward, drags the skin along with it, stretching it until one end gives way. To prevent this, the skin should be loosened from the nail once a week, not with a knife or scissors, but with something blunt, such as the end of an ivory paper cutter; this is best done after soaking the fingers in warm water, then pushing the skin back gently and slowly; the white specks on the nails are made by scraping the nail with a knife at the point where it emerges from the skin.

Biting off the finger nails is an uncleanly practice, for thus the unsightly collections at the ends are kept eaten clean! ! Children may be broken of such a filthy habit by causing them to dip the ends of their fingers several times a day in wormwood bitters, without letting them know the object; if this is not sufficient, cause them to wear caps on each finger until the practice is discontinued.

